# The effect of Sirt1 deficiency on Ca^2+^ and Na^+^ regulation in mouse ventricular myocytes

**DOI:** 10.1111/jcmm.15327

**Published:** 2020-04-27

**Authors:** Hsiang‐Yu Yang, Feng‐Zhi Lin, Hui‐Wen Yang, Pei‐Ling Yu, Shih‐Ming Huang, Yao‐Chang Chen, Chien‐Sung Tsai, Chih‐Yuan Lin

**Affiliations:** ^1^ Division of Cardiovascular Surgery Department of Surgery Tri‐Service General Hospital National Defense Medical Center Taipei Taiwan; ^2^ Grade institute of life Sciences National Defense Medical Center Taipei Taiwan; ^3^ Department of Biochemistry National Defense Medical Center Taipei Taiwan; ^4^ Department of Biomedical Engineering National Defense Medical Center Taipei Taiwan; ^5^ Department and Graduate Institute of Pharmacology National Defense Medical Center Taipei Taiwan

**Keywords:** arrhythmogenesis, Ca^2+^ and Na^+^ regulation, Sirt1

## Abstract

This study addressed the hypothesis that cardiac Sirtuin 1 (Sirt1) deficiency alters cardiomyocyte Ca^2+^ and Na^+^ regulation, leading to cardiac dysfunction and arrhythmogenesis. We used mice with cardiac‐specific *Sirt1* knockout (Sirt1^−/−^). Sirt1*^flox/flox^* mice were served as control. Sirt1^−/−^ mice showed impaired cardiac ejection fraction with increased ventricular spontaneous activity and burst firing compared with those in control mice. The arrhythmic events were suppressed by KN93 and ranolazine. Reduction in Ca^2+^ transient amplitudes and sarcoplasmic reticulum (SR) Ca^2+^ stores, and increased SR Ca^2+^ leak were shown in the Sirt1^−/−^ mice. Electrophysiological measurements were performed using patch‐clamp method. While L‐type Ca^2+^ current (*I*_Ca, L_) was smaller in Sirt1^−/−^ myocytes, reverse‐mode Na^+^/Ca^2+^ exchanger (NCX) current was larger compared with those in control myocytes. Late Na^+^ current (*I*_Na, L_) was enhanced in the Sirt1^−/−^ mice, alongside with elevated cytosolic Na^+^ level. Increased cytosolic and mitochondrial reactive oxygen species (ROS) were shown in Sirt1^−/−^ mice. Sirt1^−/−^ cardiomyocytes showed down‐regulation of L‐type Ca^2+^ channel α1c subunit (Cav1.2) and sarcoplasmic/endoplasmic reticulum Ca^2+^ ATPase 2a (SERCA2a), but up‐regulation of Ca^2+^/calmodulin‐dependent protein kinase II and NCX. In conclusions, these findings suggest that deficiency of Sirt1 impairs the regulation of intracellular Ca^2+^ and Na^+^ in cardiomyocytes, thereby provoking cardiac dysfunction and arrhythmogenesis.

## 1 INTRODUCTION

Sirtuins are nicotinamide adenine dinucleotide‐dependent class III histone deacetylases that are involved in ageing, gene silencing and DNA damage repair.^1^, ^2^, ^3^ Sirtuin 1 (Sirt1), one of the sirtuins, deacetylates a variety of substrates and modulates angiogenesis and vascular tone, thereby may provide protective effect on atherosclerosis, cardiac ischaemic/reperfusion injury, and catecholamine‐induced cardiomyopathy.^4^, ^5^, ^6^, ^7^ Sirt1 activator resveratrol was shown to reduce the degree of cardiac dysfunction and hypertrophy in spontaneously hypertensive rats.^8^ However, the underlying mechanism employed by Sirt1 in protecting cardiac health remains to be elucidate.^9^

Sirt1 deficiency may be proarrhythmogenic. It has recently been reported that Sirt1‐deficient mice exhibit abnormalities in cardiac conduction and arrhythmia‐induced premature death that leads to the hyperacetylation of Na^+^ channels.^10^ Furthermore, Sirt1 modulates intracellular Ca^2+^ homeostasis.^11^, ^12^, ^13^ Recently a study reported deficiency of Sirt1 enhances the acetylation and alters the function of sarcoplasmic/endoplasmic reticulum Ca^2+^ ATPase (SERCA).^14^ However, the correlation between Sirt1 and Ca^2+^ handling and the effect of Sirt1 deficiency on the homeostasis of intracellular Ca^2+^ and Na^+^ in cardiomyocytes are complex and remain poorly understood.

The aim of the present study was to assess whether Sirt1 deficiency in the heart of mice alters intracellular Ca^2+^ and Na^+^ regulation, resulting in cardiac dysfunction and predisposition to arrhythmia. Here, we used cardiac‐specific *Sirt1* knockout mice to identify the Ca^2+^ and Na^+^ regulatory mechanisms affected. We assessed a variety of indices that mandate cardiac function and ionic regulation to establish a clear picture of pathological cellular Ca^2+^ and Na^+^ homeostasis in the heart with Sirt1 deficiency. Our results suggest deficiency of cardiac Sirt1 promote dysregulation of Ca^2+^ and Na^+^, leading to contractile dysfunction and providing proarrhythmic substrates.

## 2 METHODS

### 2.1 Genetically modified mice models

Animal experiments were all conducted with the approval of the Institutional Animal Care and Use Committee (IACUC 18‐056) of the National Defense Medical Center, Taipei, Taiwan and in accordance with the National Institutes of Health guidelines, ‘Guide for the Care and Use of Laboratory Animals’, on the operation of experimental animals.

Mice with Cardiac‐specific *Sirt1* exon 4 knockout (Sirt1^−/−^) were created by crossing Sirt1*^flox/flox^* mice (Sirt1*^flox/flox^* was the control mice that were purchased from Jackson Laboratory) with α‐MHC (myosin heavy chain) promoter‐driven Cre mice with C57BL/6J background (α‐MHC‐Cre, courtesy of Professor M. Schneider, Imperial College London) and are currently in use in the laboratory.^15^ Male Sirt1*^flox/flox^* (control) and Sirt1^−/−^ 40‐week‐old mice were killed, and the hearts were procured for subsequent experiments. Animals were kept at temperature of 21 ± 1°C under controlled 12:12 h light‐dark lighting cycle with ad libitum access to standard chow (0.28% [w/w] NaCl, 1.00% [w/w] CaCl_2_, 0.22% [w/w] MgCl_2_; LabDiet, USA) and deionized drinking water before use.

### 2.2 Echocardiography

A Mindray M9 ultrasound machine (Mindray Co, Shen Zhen, China) equipped with a 12MHz probe was used to measure the cardiac functional changes in the experimental mice. Mice were subjected to echocardiography under anaesthesia with ketamine (100 mg/kg, intraperitoneal) and xylazine (5 mg/kg, intraperitoneal) during echocardiography. In short‐axis view, M‐mode traces were obtained to measure left ventricle (LV) wall thickness and chamber dimensions at diastole and systole and echocardiography‐calculated LV mass. The Teichholz formula was used to calculate LV volumes: 7/ (2.4 + D) × D^3^ (D = linear LV diameter). LV ejection fraction (EF) was calculated as following equation: EF = (LV end‐diastolic volume ‐ LV end‐systolic volume)/LV end‐diastolic volume and expressed in %. Internal diameter of LVs at systolic (LVIDs) and diastolic (LVIDd) phase were recorded for calculating fractional shortening (FS) as following equation: FS = (LVIDd ‐ LVIDs)/LVIDd. The average was calculated from measurements taken from three consecutive cardiac cycles.

### 2.3 Preparation of ventricle tissues for electromechanical and pharmacological analyses

Mice were anesthetized by intraperitoneal injections of Zoletil 50 (5 mg/kg) and xylazine (5 mg/kg) with isoflurane inhalation (5% in oxygen) in a vaporizer. The hearts were harvested from the mice by performing a midline thoracotomy as described previously.^16^ The ventricular tissues were separated from the atria at the atrioventricular groove in normal Tyrode's (NT) solution. The ventricular tissue preparation was pinned with needles onto the bottom of a tissue bath. The other end part of the preparation was connected to a Grass FT03C force transducer with silk thread. The preparations were superfused with a solution composed (in mM) of 137 NaCl, 4 KCl, 15 NaHCO_3_, 0.5 NaH_2_PO_4_, 0.5 MgCl_2_, 2.7 CaCl_2_ and 11 dextrose at a constant rate (3 ml/min), saturated with a 97% O_2_ ‐ 3% CO_2_ gas mixture. The bath temperature was maintained at 37°C. Before the electrophysiological assessments, the preparations were allowed to equilibrate in the bath for 1 h.

Transmembrane action potentials (APs) were recorded using 3M KCl‐filled glass microelectrodes connected to a WPI Duo 773 electrometer as described previously.^17^ Signals were recorded digitally using a data acquisition system with a cut‐off frequency of 10‐kHz low‐pass filter and a 16‐bit accuracy at a rate of 125 kHz. Pulse stimulation with 1‐ms duration was provided by a Grass S48 stimulator through a Grass SIU5B stimulus unit. The AP durations (APDs) were measured in ventricle preparations under 2 Hz pulse stimulation. The AP amplitude (APA) was determined by the difference between the peak potential of depolarization and the resting membrane potential (RMP). The repolarization extents of 20%, 50% and 90% of the APA were denoted as the APD_20_, APD_50_ and APD_90_. Spontaneous electrical activity and arrhythmia, including burst firing, delayed after depolarizations (DADs), and ventricular tachycardia were recorded and analysed. Ventricle preparations were perfused with KN93, a calmodulin‐dependent protein kinase II (CaMKII) inhibitor, (1 μmol/L) or ranolazine,^18^ a selective late Na^+^ current (*I*_Na,L_) inhibitor, (10 μmol/L) at a constant rate to determine pharmacological responses.

### 2.4 Cardiomyocyte isolation

Ventricular myocytes were enzymatically dissociated as previously described with modifications.^19^ Briefly, mice were killed using a mixture of Zoletil 50 and xylazine, and the hearts were procured and cannulated via the aorta to a Langendorff perfusion system at 37°C. The heart was firstly perfused with normal Tyrode's (NT) solution for 10 minutes and digested with Ca^2+^‐free solution containing 1 mg/mL collagenase (type I; Sigma‐Aldrich, St. Louis, MO, USA) and 0.06 mg/mL proteinase (type XIV; Sigma‐Aldrich, St. Louis, MO, USA). After perfusion, the heart was taken down from the cannula, cut into small pieces, gently triturated with a plastic transfer pipette and filtered through a nylon mesh. The dissociated cells were stored in NT at 20‐22°C. Rod‐shaped cells with clear striations and no granulation were used within 6‐8 hours for all the experiments.

### 2.5 Composition of solutions

#### 2.5.1 Normal Tyrode's solution

Tyrode's solution contained 137 mmol/L NaCl, 1.8 mmol/L CaCl_2_, 0.5 mmol/L MgCl_2_, 5.4 mmol/L KCl, 10 mmol/L glucose and 10 mmol/L 4‐(2‐Hydroxyethyl)piperazine‐1‐ethanesulfonic acid (HEPES) (pH adjusted to 7.4 with NaOH).

#### 2.5.2 Ca^2+^‐free solution

Ca^2+^‐free solution comprised 120 mmol/L NaCl, 5.4 mmol/L KCl, 1.2 mmol/L MgSO_4_, 1.2 mmol/L KH_2_PO_4_, 6 mmol/L HEPES, 10 mmol/L glucose and 10 mmol/L taurine (pH adjusted to 7.4 using NaOH).

#### 2.5.3 Micropipettes solution

Micropipettes solution for *I*_Ca,L_ was composed of 130 mmol/L CsCl, 1 mmol/L MgCl_2_, 5 mmol/L Mg ATP, 10 mmol/L HEPES, 0.1 mmol/L NaGTP and 5 mmol/L Na_2_‐phosphocreatine (pH adjusted to 7.2 with CsOH). For nickel‐sensitive Na^+^/Ca^2+^ exchanger (NCX) current, the solution comprised 20 mmol/L NaCl, 110 mmol/L CsCl, 0.4 mmol/L MgCl_2_, 20 mmol/L TEACl, 1.75 mmol/L CaCl_2_, 5 mmol/L 1,2‐Bis(2‐aminophenoxy)ethane‐N,N,N′,N′‐tetraacetic acid (BAPTA), 5 mmol/L Mg ATP, 5 mmol/L glucose and 10 mmol/L HEPES (pH adjusted to 7.25 using CsOH). For the *I*_Na_, the solution was composed of 133 mmol/L CsCl, 5 mmol/L NaCl, 10 mmol/L ethylene glycol tetraacetic acid (EGTA), 5 mmol/L Mg_2_ ATP, 20 mmol/L TEACl and 5 mmol/L HEPES (pH adjusted to 7.3 with CsOH). For *I*_Na,L_, the solution was composed of 130 mmol/L CsCl, 4 mmol/L Na_2_ ATP, 10 mmol/L EGTA, 1 mmol/L MgCl_2_ and 5 mmol/L HEPES (pH adjusted to 7.3 with NaOH).

#### 2.5.4 External solution

The external solution for NCX experiment contained 140 mmol/L NaCl, 2 mmol/L CaCl_2_, 1 mmol/L MgCl_2_, 5 mmol/L HEPES and 10 mmol/L glucose with 10 μmol/L strophanthidin to block the Na^+^/K^+^ pump, and 10 μmol/L nitrendipine and 100 μmol/L niflumic acid to block Ca^2+^‐activated Cl^−^ currents (pH adjusted to 7.4 with NaOH). For *I*_Na_, the solution contained 5 mmol/L NaCl, 133 mmol/L CsCl, 2 mmol/L MgCl_2_, 1.8 mmol/L CaCl_2_, 0.002 mmol/L nifedipine, 5 mmol/L glucose and 5 mmol/L HEPES (pH adjusted to 7.3 with NaOH). For *I*_Na,L_, the solution contained 130 mmol/L NaCl, 5 mmol/L CsCl, 1 mmol/L MgCl_2_, 1 mmol/L CaCl_2_, 10 mmol/L glucose and 10 mmol/L HEPES (pH adjusted to 7.4 with NaOH). For sarcoplasmic reticulum (SR) Ca^2+^ leak, the 0 Na^+^/0 Ca^2+^ solution had the same composition with NT but no added Ca^2+^, 10 mmol/L EGTA and 140 mmol/L LiCl substituted for NaCl (pH adjusted to 7.4 with LiOH).

### 2.6 Intracellular Ca^2+^ monitoring

Cardiomyocytes from control and Sirt1^−/−^ mice were loaded with Ca^2+^ dye (10 μmol/L Fluo‐3 AM) at room temperature for 30 minutes and imaged as previously described method.^20^, ^21^ Briefly, fluorescence microscopy was performed using an inverted laser‐scanning confocal microscope (Zeiss LSM 510; Carl Zeiss, Jena, Germany). The fluorescent signals (*F*) were normalized against the baseline fluorescence (*F*_0_) to obtain reliable information about transient intracellular Ca^2+^ changes (Ca^2+^ transient = [*F* − *F*_0_]/*F*_0_) and to correct the variations in the fluorescence intensity due to different amount of dye uptake into cells. The Ca^2+^ transient was measured with 1‐Hz field stimulation. After achieving a steady‐state Ca^2+^ transients with the repeated pulses (1 Hz for 15 seconds), the superfusate was rapidly switched to 0 Na^+^/0 Ca^2+^ solution with 1 mmol/L tetracaine for a minimum of 20 seconds. The SR Ca^2+^ leak was measured as the tetracaine (1 mmol/L)‐reduced intracellular Ca^2+^ as previously described.^22^ The SR Ca^2+^ stores were assessed by rapid application of 20 mmol/L caffeine after a pule stimulation train at 1 Hz for 30 seconds. The SR Ca^2+^ stores were estimated from the peak amplitudes of the caffeine‐provoked Ca^2+^ transient. The integral of the inward NCX current induced by fast application of 20 mmol/L caffeine to cells voltage‐clamped at −40 mV was used to calculate SR Ca^2+^ content as previously described,^23^ which was determined using the equation: SR Ca^2+^ content (μmol/L/L cytosol) = [(1 + 0.12) × *C*_caff_/F × 1000)]/ (*C*_m_ × 8.31 × 8.44), where *C*_caff_ is the integral of the inward NCX current induced by caffeine, *F* is Faraday's number, *C*_m_ is the membrane capacitance, and cell surface‐to‐volume ratio was 8.44 pF/pL.

### 2.7 Electrophysiological measurement

#### 2.7.1 *I*_Ca,L_, NCX current, *I*_Na_, and *I*_Na,L_

Electrophysiological properties of ventricular myocytes were obtained by whole‐cell configuration patch‐clamp techniques with Axopatch 1D amplifier (Axon Instruments, Foster city, USA) as described previously.^16^ A small hyperpolarizing pulse from a holding potential of −50 mV to a potential of −55 mV for 80 ms was delivered at the beginning of each experiment. The area under the capacitive current was divided by the applied voltage step to obtain the cell capacitance. Series resistance was electronically compensated about 60%‐80%. *I*_Ca,L_ was determined as an inward current during voltage‐clamp steps from a holding potential of −50 mV to potentials from −40 to +60 mV in 10‐mV steps for 300 ms at a frequency of 0.1 Hz. *I*_Ca,L_ was assessed between 5‐15 minutes after membrane patch rupture in each cardiomyocyte to avoid ‘run‐down’ effects. The current of NCX was measured using voltage‐clamp potentials between −100 and +100 mV from a holding potential of −40 mV in 20‐mV steps for 300 ms at a frequency of 0.1 Hz. NCX current amplitudes were determined as Nickel (10 mmol/L NiCl_2_)‐sensitive currents as previously described.^21^ The *I*_Na_ was elicited during potential steps from a holding potential of −120 mV to testing potentials from −80 to 0 mV in 10‐mV steps for 40 ms at a frequency of 3 Hz. *I*_Na,L_ was measured using a step/ramp protocol as described below: start with a potential of −100 mV stepping to +20 mV for 100 ms afterwards ramp back to −100 mV for 100 ms The *I*_Na,L_ was determined as tetrodotoxin (30 µmol/L TTX)‐sensitive current obtained when the potential was ramped back to −100 mV.

### 2.8 Measurement of ROS and cytosolic Na^+^ level

Ventricular myocytes were incubated in NT solution with 10 μmol/L CellROX green and 2 μmol/L MitoSOX Red (Life Technologies) to assess cytosolic and mitochondria reactive oxygen species (ROS) production, respectively. Myocytes incubated with 5 μmol/L Asante NaTRIUM Green‐2 AM (Teflabs) was used to measure the cytosolic Na^+^ level. Experiments were conducted using an inverted laser‐scanning confocal microscope (Zeiss LSM 510, Carl Zeiss) with a 63x1.25 objective as previously described.^24^ Excitation light with wavelength of 488 nm was used, and emission fluorescence was detected at wavelengths over 505 nm in the XY mode of the confocal microscope system. Cardiac myocytes were paced at 1 Hz in the experiment. Images were analysed using ImageJ as described previously.^25^

### 2.9 Western blot analysis

The protein extraction buffer contained 100 mmol/L Tris‐HCl (pH 8.0), 0.1% sodium dodecyl sulphate, 1% Triton X‐100, 150 mmol/L NaCl and protease inhibitor cocktail (Roche). The cardiac protein extracts were separated by sodium dodecyl sulphate polyacrylamide gel electrophoresis and then transferred to polyvinylidene difluoride membranes (Millipore) that were incubated with the listed antibodies: Cav1.2 (1:1000, AACC‐033, rabbit polyclonal antibody; Alomone Labs, Jerusalem, Israel), CaMKII (1:1000, sc‐5306, mouse monoclonal antibody; Santa Cruz Biotechnology, Dallas, USA), NCX (1:1000, mouse monoclonal antibody, ab2869; Abcam), SERCA2a (1:5000, sc‐376235, mouse monoclonal antibody; Santa Cruz Biotechnology, Dallas, USA) and α‐tubulin (1:10 000, sc‐5286, mouse monoclonal antibody; Santa Cruz Biotechnology, Dallas, USA). Subsequently, the membranes were incubated with anti‐mouse (sc‐2056; Santa Cruz Biotechnology, Dallas, USA or anti‐rabbit (sc‐2004; Santa Cruz Biotechnology, Dallas, USA) secondary IgG antibodies at a dilution of 1:10 000. Immunoreactive proteins were detected by enhanced chemiluminescence (GE Healthcare, Chicago, USA) and quantified using the ImageJ software.

### 2.10 Acquisition systems and statistical analysis

Continuous values have been expressed as mean ± SEM. Student's *t* test, or Pearson's chi‐square test were used to compare the differences. The SigmaPlot version 12 (Systat Software Inc., San Jose, CA, USA) was used for statistical comparisons. The ‘n’ stands for the total cells from the total number of hearts (n = cells/hearts) and the ‘N’ is the animal numbers. Statistical significance was represented as *, **, and *** for *P* < 0.05, *P* < 0.01 and *P < *0.005, respectively.

## 3 RESULTS

### 3.1 In Vivo M‐mode echocardiography

**FIGURE 1:**
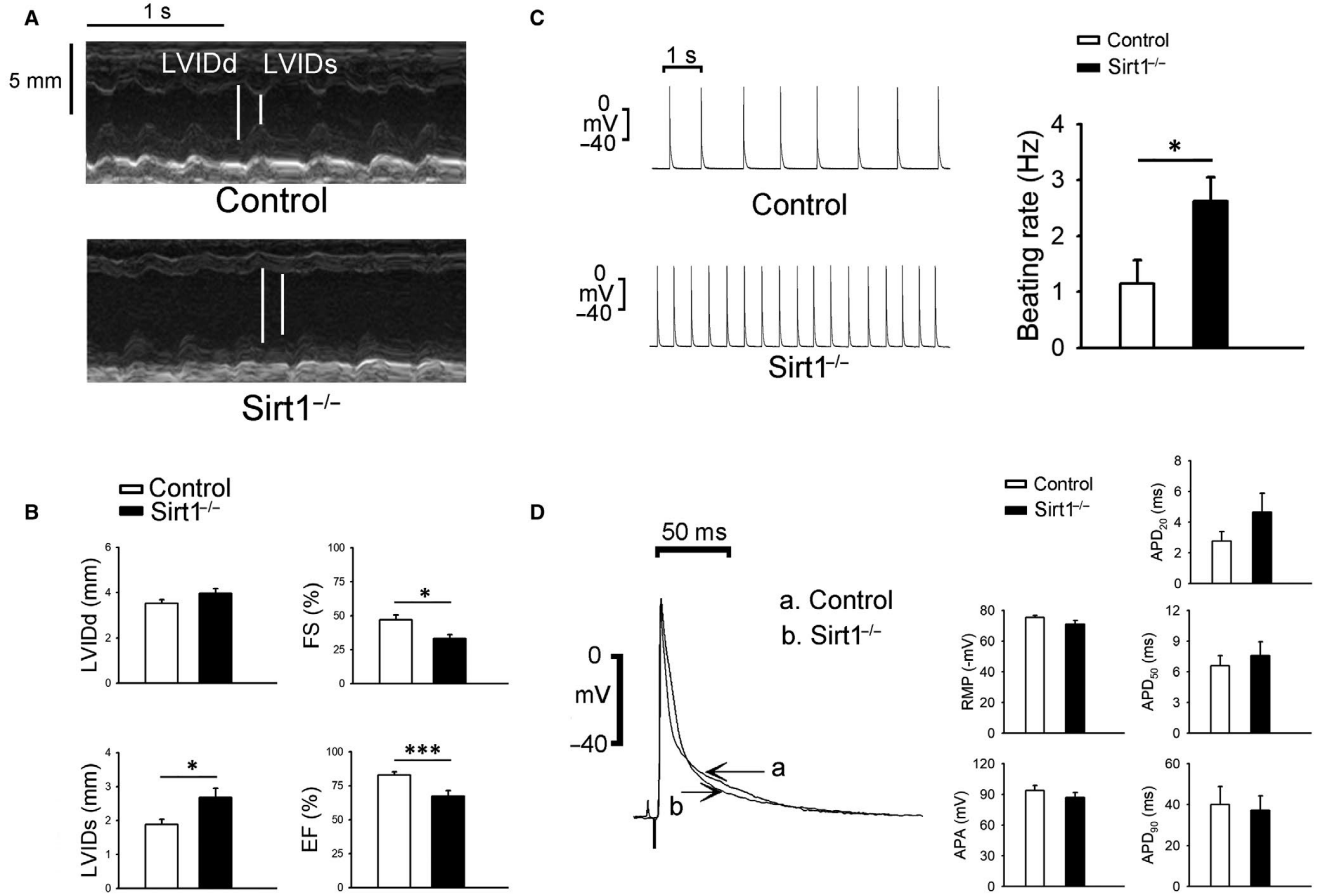
Echocardiograms and action potentials of ventricles in control and Sirt1^−/−^ mice. A, Representative in vivo M‐mode echocardiograms for LVIDd and LVIDs in control and Sirt1^−/−^ mice. B, Mean data for LVIDd, LVIDs, FS and EF (Control N = 6 and Sirt1^−/−^ N = 6; *P < 0.05, ***P < 0.005). C, Representative recordings of spontaneous potentials and mean data for beating rate in the ventricles of Sirt1^−/−^ and control mice (Control N = 6 and Sirt1^−/−^ N = 6; *P < 0.05). D, Average RMP, APA, APD_20_, APD_50_ and APD_90_ in Sirt1^−/−^ and control mice ventricular preparations (Control N = 6 and Sirt1^−/−^ N = 6). APA, action potentials amplitude; APD, action potentials durations; EF, ejection fraction; FS, fractional shortening; LVIDd, Internal diameter of left ventricles at diastolic; LVIDs, Internal diameter of left ventricles at systolic; RMP, resting membrane potential

Sirt1^−/−^ mice possessed larger LVIDs than those in the control group (Figure 1B). FS and EF decreased in the Sirt1^−/−^ mice as compared to those in the control mice (Figure 1B).

### 3.2 Ventricle electrical activity

**FIGURE 2:**
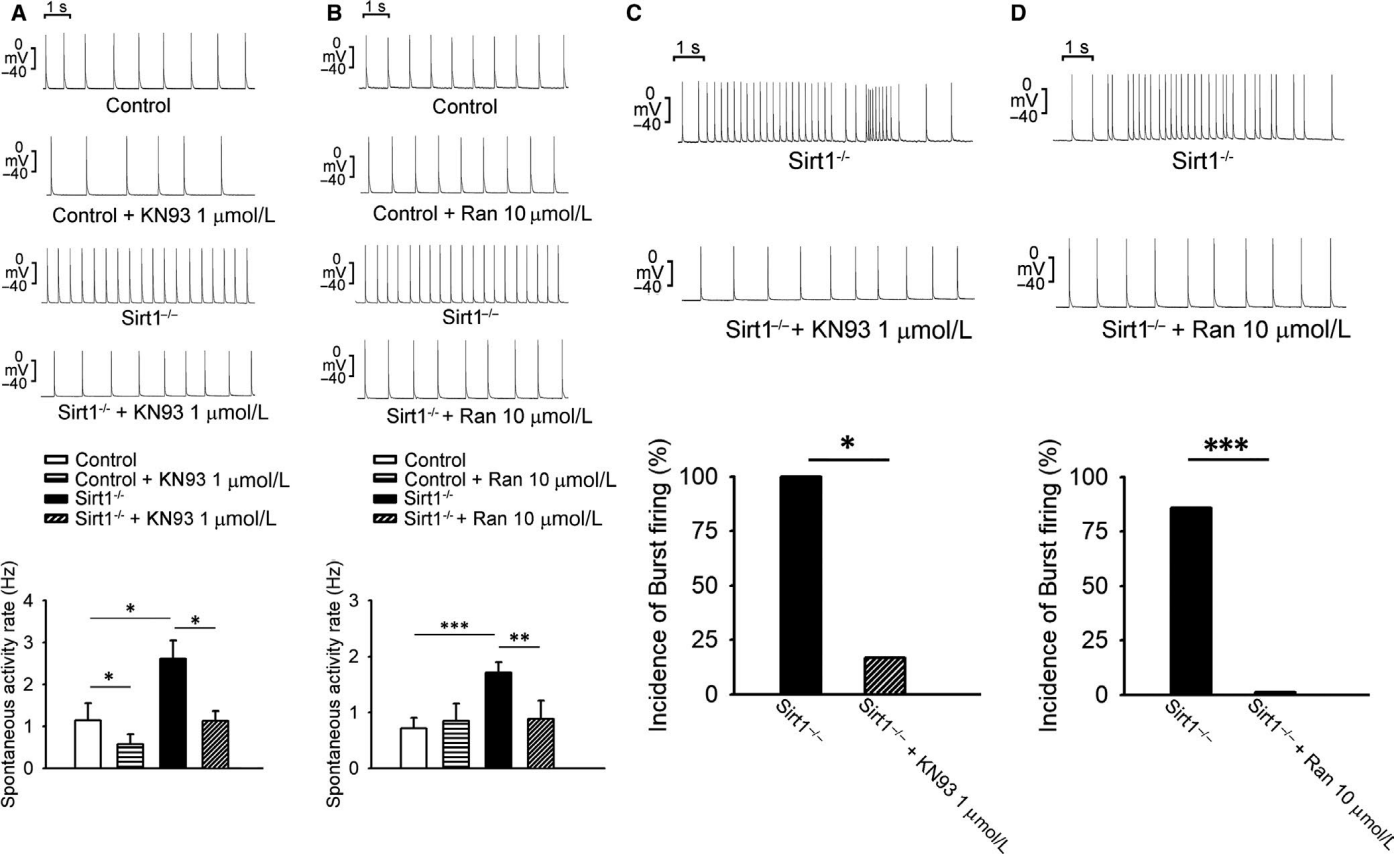
Electrocardiographic changes of ventricles and burst firing in control and Sirt1^−/−^ mice. A and B, Sirt1^−/−^ ventricles showed faster rates of spontaneous activity as compared to those in the control mice that were suppressed upon treatment with KN93 (Control N = 6 and Sirt1^−/−^ N = 6; *P < 0.05) and ranolazine (Control N = 6 and Sirt1^−/−^ N = 7; **P < 0.01, ***P < 0.005). C and D, Sirt1^−/−^ ventricles showed an increased incidence of burst firing that was inhibited by KN93 and ranolazine (Sirt1^−/−^ N = 7; *P < 0.05, ***P < 0.005)

The ventricles in Sirt1^−/−^ mice showed faster rates of spontaneous activity as compared with those in the control mice (Figure 1C). The APD_20_, APD_50_, APD_90_, APA and RMP showed no difference between the Sirt1^−/−^ and control mice (Figure 1D). The increase in the rate of spontaneous activity in the ventricles of Sirt1^−/−^ mice was suppressed by KN93, and ranolazine (Figure 2A,B). Furthermore, Sirt1^−/−^ ventricles showed an increased incidence of burst firing compared with that in the control mice; this phenotype was abrogated upon treatment with KN93 or ranolazine (Figure 2C,D).

### 3.3 Ca^2+^ transient amplitudes, SR Ca^2+^ stores and SR Ca^2+^ leak

**FIGURE 3:**
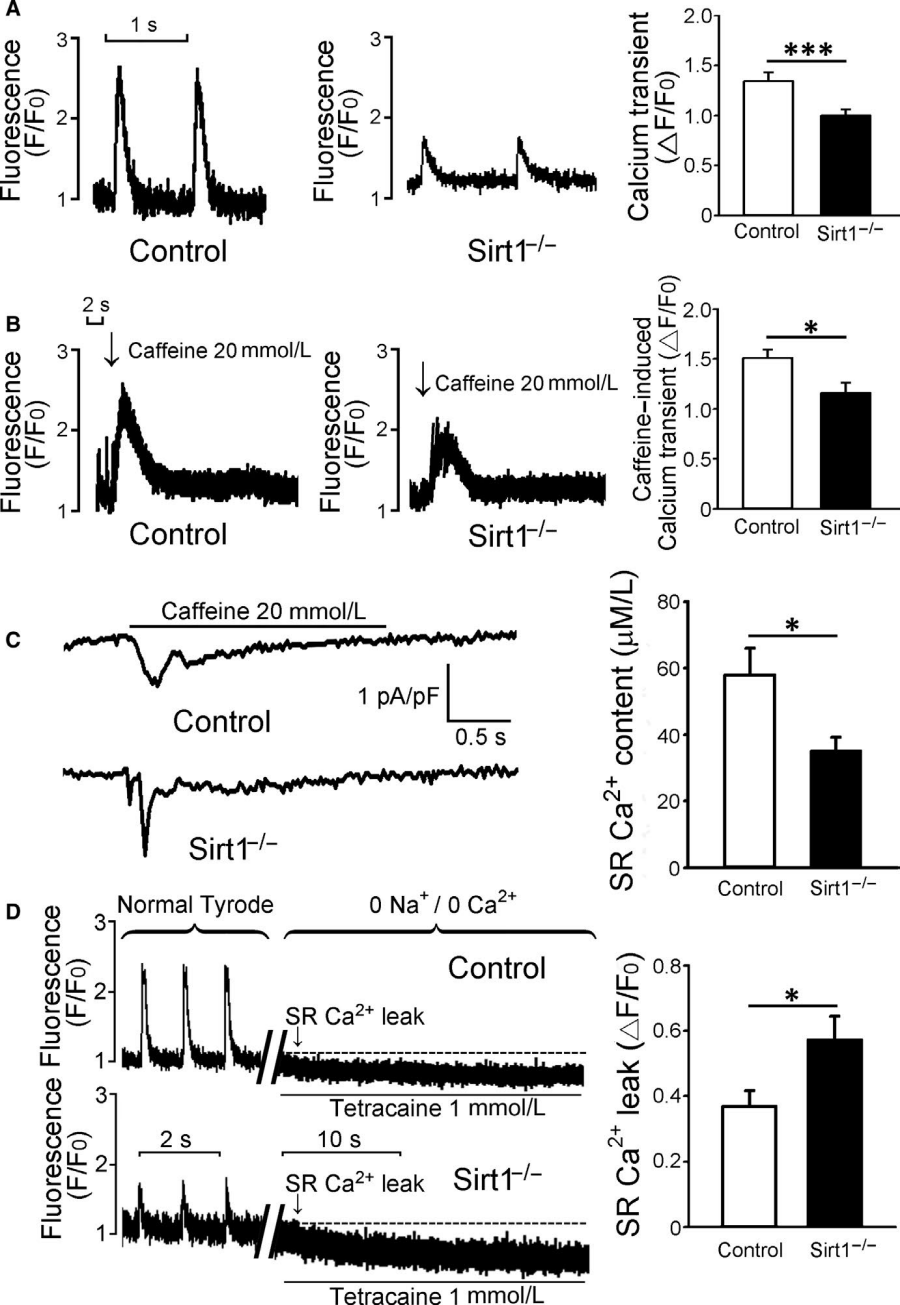
Ca^2+^ transient, SR Ca^2+^ stores and SR Ca^2+^ leak. A, Representative traces of steady‐state Ca^2+^ transients of control and Sirt1^−/−^ cardiomyocytes loaded with Fluo‐3. Cardiomyocytes from Sirt1^−/−^ mice showed lower Ca^2+^ transient amplitudes as compared to those in the control mice (Control n = 56/6 and Sirt1^−/−^ n = 56/7; ***P < 0.005). B, Typical traces of caffeine‐induced transient amplitudes of Ca^2+^ in control and Sirt1^−/−^ cardiomyocytes. Sirt1^−/−^ cardiomyocytes exhibited lower caffeine‐induced Ca^2+^ transient amplitudes compared with those from the control mice (Control n = 31/6 and Sirt1^−/−^ n = 22/7; *P < 0.05). C, Typical traces of caffeine‐induced Na^+^/Ca^2+^ exchanger inward current in control and Sirt1^−/−^ myocytes. Sirt1^−/−^ cardiomyocytes had lower SR Ca^2+^ content as compared to the control mice (Control n = 11/3 and Sirt1^−/−^ n = 10/3; *P < 0.05). D, Typical recordings of SR Ca^2+^ leak determined by fast tetracaine application in control and Sirt1^−/−^ cardiomyocytes. Sirt1^−/−^ cardiomyocytes had more Ca^2+^ leakage from SR compared to the control cardiomyocytes (Control n = 15/3 and Sirt1^−/−^ n = 22/3; *P < 0.05). SR, sarcoplasmic reticulum

Steady‐state and caffeine‐induced Ca^2+^ transient amplitudes in cardiomyocytes were 26% and 23% lesser in Sirt1^−/−^ mice as compared with those in the control mice, respectively (Figure 3A,B). Sarcoplasmic reticulum Ca^2+^ content, obtained by integrating the caffeine‐induced inward NCX current, was 39% less in the Sirt1^−/−^ mice than that in the control mice (Figure 3C). Sirt1^−/−^ cardiomyocytes had 55% larger SR Ca^2+^ leak compared with that in the control cardiomyocytes (Figure 3D).

### 3.4 L‐type Ca^2+^ current and nickel‐sensitive NCX current

**FIGURE 4:**
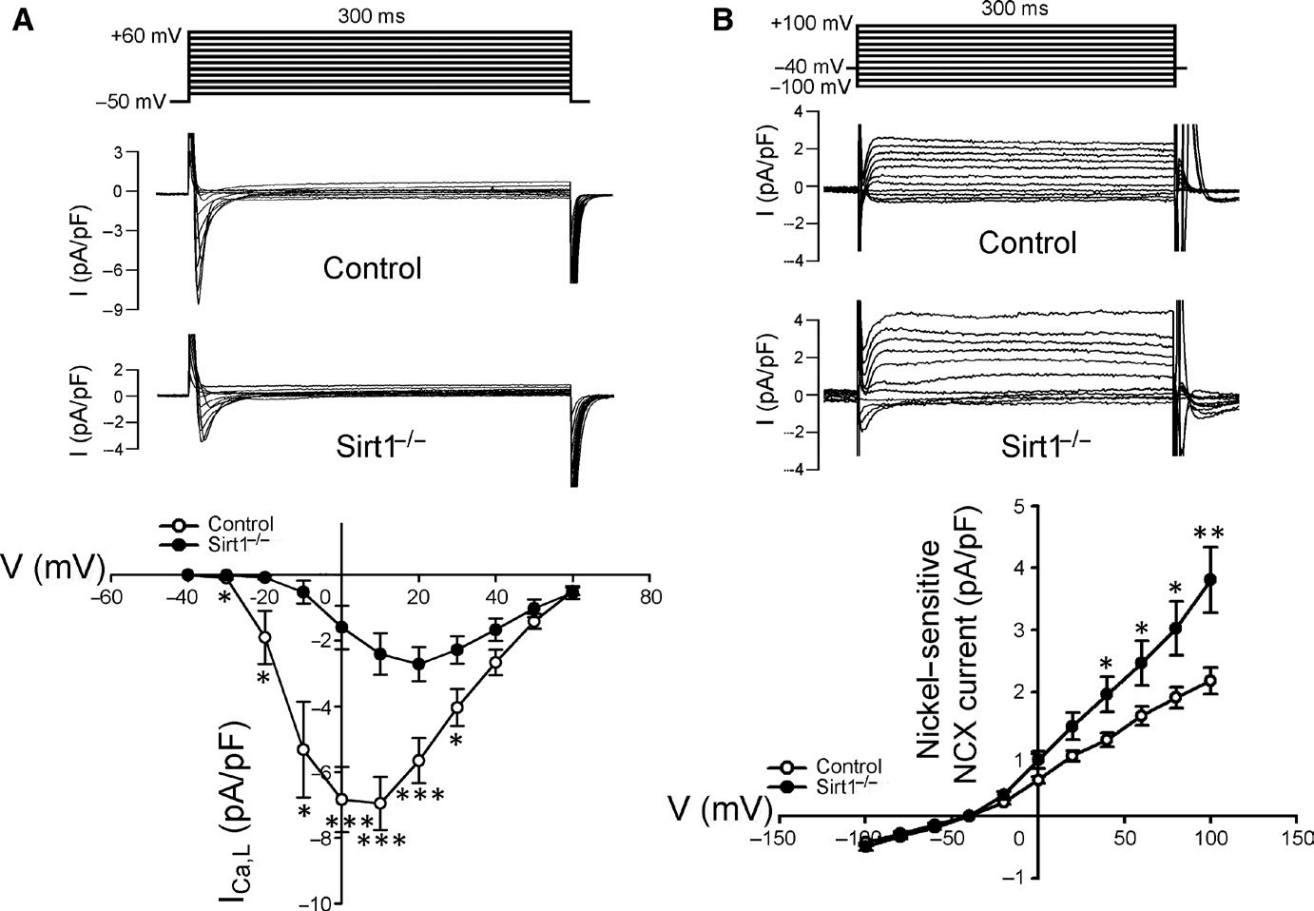
*I*_Ca,L_ and NCX current. A, Voltage‐clamp protocol with corresponding traces of *I*_Ca,L_ and current‐voltage (*I*‐*V*) relationships between control and Sirt1^−/−^ myocytes (Control n = 12/4 and Sirt1^−/−^ n = 9/3; *P < 0.05, ***P < 0.005). B, the voltage‐clamp protocol with the corresponding traces of nickel‐sensitive NCX current and the *I*‐*V* relationships from control and Sirt1^−/−^ myocytes (Control n = 9/4 and Sirt1^−/−^ n = 10/4; *P < 0.05, ***P < 0.005). NCX, Na^+^/Ca^2+^ exchanger

The density of *I*_Ca,L_ in the Sirt1^−/−^ myocytes was smaller compared with those in the control myocytes (Figure 4A). Moreover, Sirt1^−/−^ ventricular myocytes showed larger reverse‐mode of nickel‐sensitive NCX current compared with that in the control ventricular myocytes (Figure 4B).

### 3.5 *I*_Na_, *I*_Na,L_, and cytosolic Na^+^ levels

**FIGURE 5:**
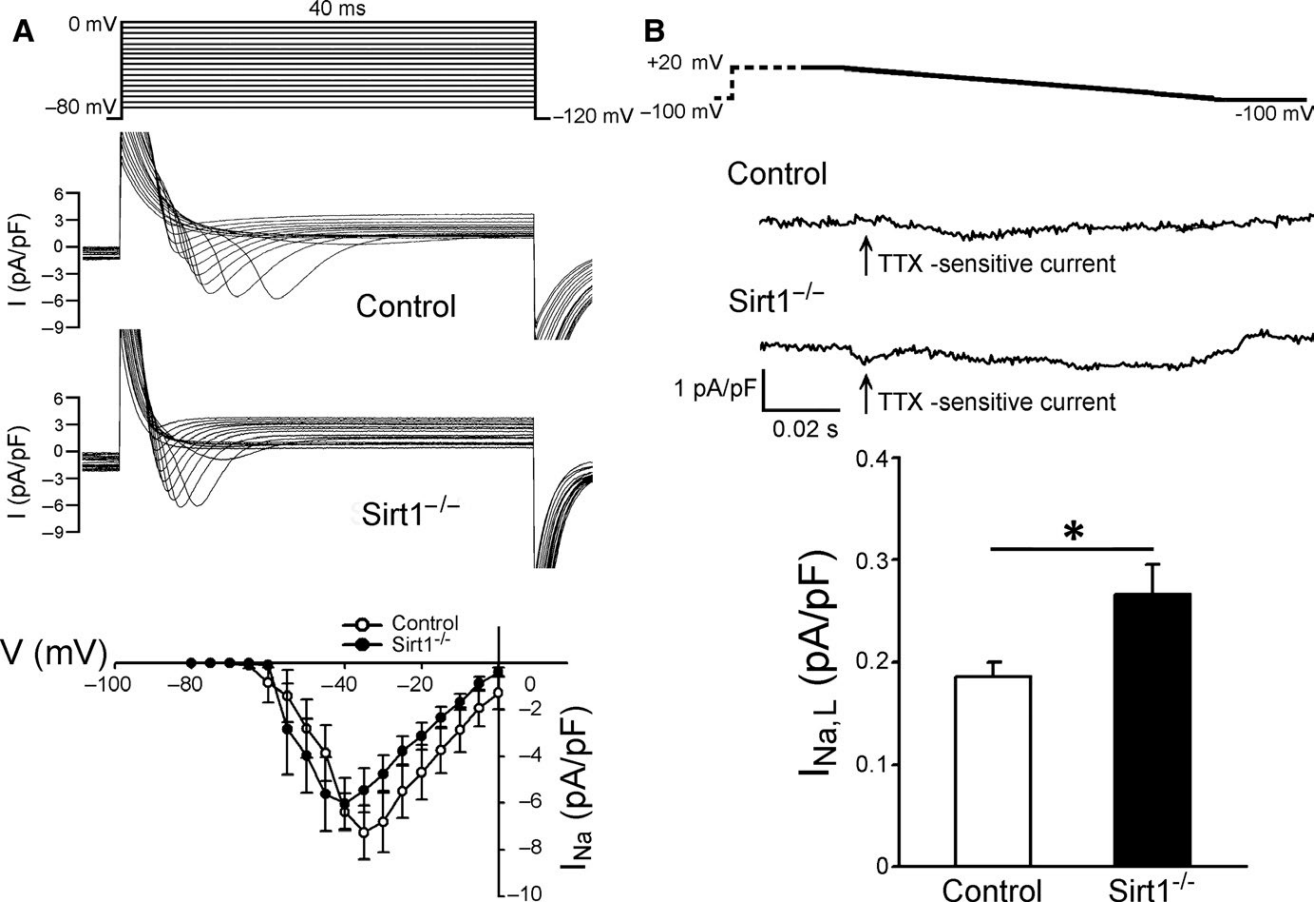
*I*_Na_, *I*_Na,L_ and cytosolic Na^+^ levels. A, Representative figures for the voltage‐clamp protocol with corresponding traces of *I*_Na_ and *I*‐*V* relationships between control and Sirt1^−/−^ myocytes (Control n = 13/3 and Sirt1^−/−^ n = 12/3). B, Representative figures for the voltage‐clamp protocol with corresponding traces of tetrodotoxin (TTX)‐sensitive current (*I*_Na,L_) and *I*‐*V* relationships between control and Sirt1^−/−^ myocytes (Control n = 10/4 and Sirt1^−/−^ n = 10/3; *P < 0.05)

**FIGURE 6:**
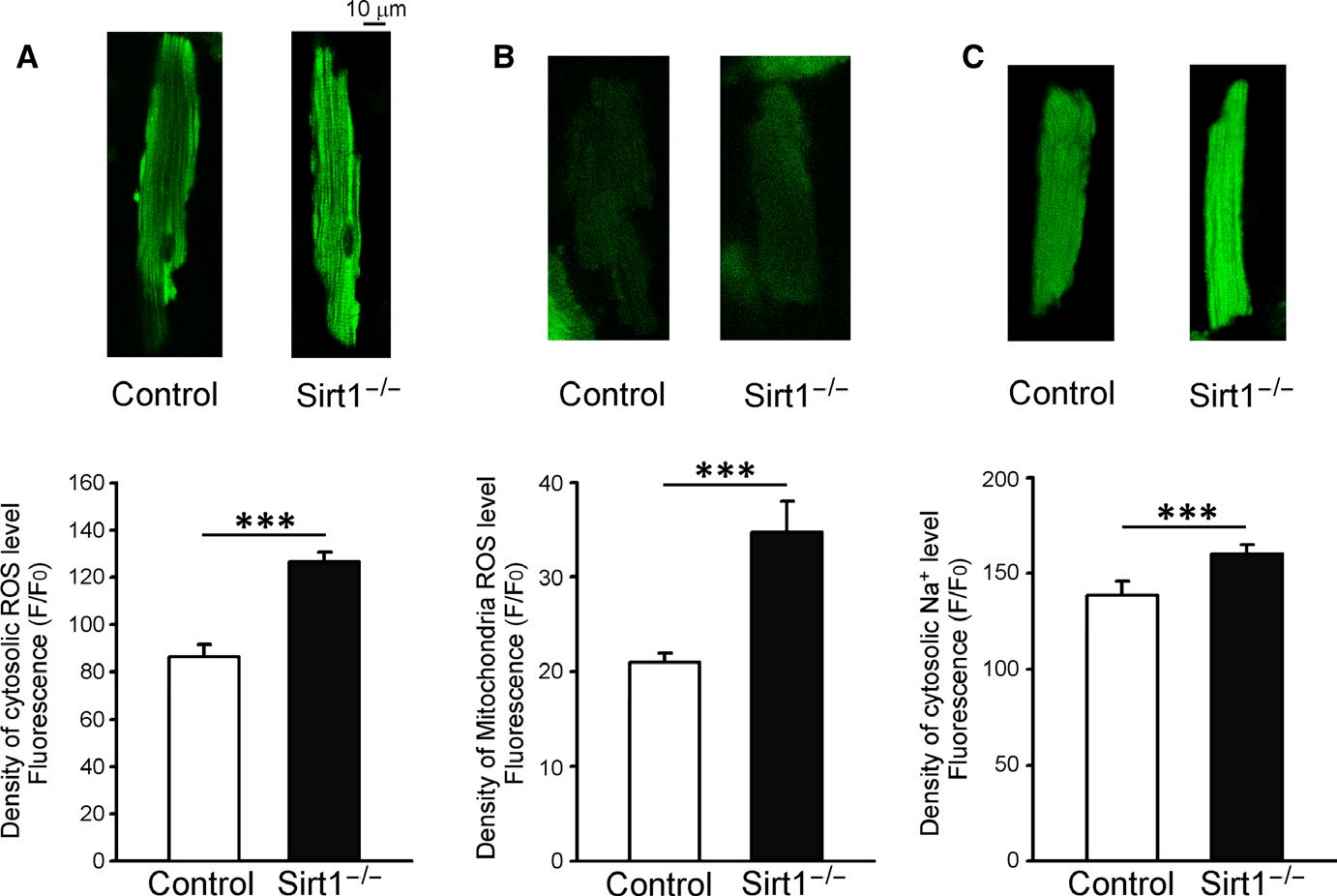
Oxidative stress and cytosolic Na^+^ levels in control and Sirt1^−/−^ ventricular myocytes. A, Typical fluorescent images and mean data for the cytosolic levels of reactive oxygen species (ROS) in control and Sirt1^−/−^ ventricular myocytes (Control n = 29/6 and Sirt1^−/−^ n = 26/5; ***P < 0.005). B, Typical fluorescent images and mean data for the levels of ROS in the mitochondria in control and Sirt1^−/−^ ventricular myocytes (Control n = 29/3 and Sirt1^−/−^ n = 31/3; ***P < 0.005). C, Typical fluorescent images and mean data for the cytosolic levels of Na^+^ in control and Sirt1^−/−^ ventricular myocytes (Control n = 34/5 and Sirt1^−/−^ n = 37/5; ***P < 0.005)

While *I*_Na_ current density was not different in the Sirt1^−/−^ and control myocytes (Figure 5A), the current density of *I*_Na, L_ (tetrodotoxin‐sensitive current) in the Sirt1^−/−^ myocytes was greater than that in the control myocytes (0.27 ± 0.03 and 0.18 ± 0.02 pA/pF, respectively; *P < 0.05; Figure 5B). Moreover, the intracellular Na^+^ concentration ([Na^+^]*_i_*) in the Sirt1^−/−^ cardiomyocytes was higher compared with that in the control cardiomyocytes (157 ± 33 *F*/*F*_0_, n = 24/3 and 100.6 ± 6.8 *F*/*F*_0_, n = 24/3, respectively; ***P < 0.005; Figure 6C).

### 3.6 Oxidative stress

Sirt1^−/−^ ventricular myocytes had higher levels of cytosolic ROS compared with those in the control ventricular myocytes (122.2 ± 5.0 *F*/*F*_0_, n = 23/5 and 90.1 ± 4.8 *F*/*F*_0_, n = 40/5, respectively; ***P < 0.005; Figure 6A). Mitochondrial ROS was higher in Sirt1^−/−^ ventricular myocytes as compared to that in the control ventricular myocytes (35.0 ± 4.823 *F*/*F*_0_, n = 16/3 and 22.5 ± 1.1 *F*/*F*_0_, n = 18/3, respectively; ***P < 0.005; Figure 6B).

### 3.7 Expression of intracellular Ca^2+^ regulatory proteins

**FIGURE 7:**
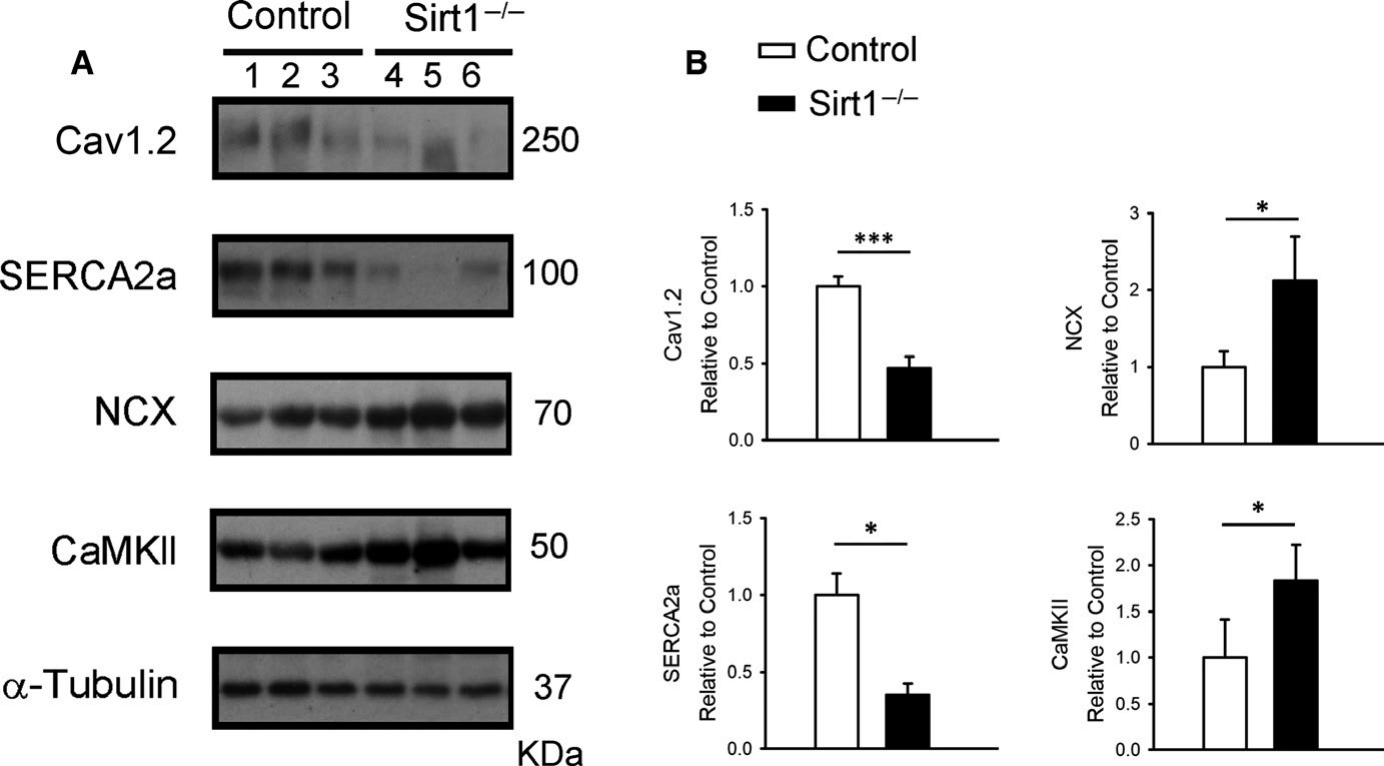
Ca^2+^ regulatory proteins in control and Sirt1^−/−^ ventricular myocytes. A, Representative immunoblot and mean data for Cav1.2, SERCA2a, NCX and CaMKII in control and Sirt1^−/−^ mice ventricular myocytes. B, Normalized densitometry for the protein levels (α‐Tubulin was used as an internal control; Control N = 6 and Sirt1^−/−^ N = 6; *P < 0.05)

We determined the protein expressions associated with intracellular Ca^2+^ regulation in the cardiomyocytes of control and Sirt1^−/−^ mice using Western blotting (Figure 7A). The L‐type Ca^2+^ channel subunit α1c was down‐regulated in Sirt1^−/−^ ventricles compared with the control ventricles (Figure 7B). While the protein levels of SERCA2a was reduced in Sirt1^−/−^ mice, NCX and CaMKII in Sirt1^−/−^ ventricles were up‐regulated compared with the control mice (Figure 7B).

## 4 DISCUSSION

This study demonstrated that the deficiency of cardiac Sirt1 alters the regulation of Ca^2+^ and Na^+^ in cardiomyocytes and stimulates arrhythmia. Sirt1^−/−^ mice showed cardiac dysfunction, enhanced ventricular arrhythmia, impaired Ca^2+^ handling and Na^+^ regulation and increased ROS production. These results suggest that Sirt1 deficiency induces proarrhythmia and cardiac dysfunction by altering Ca^2+^ and Na^+^ homeostasis in ventricular myocytes.

Sirt1^−/−^ cardiomyocytes had smaller Ca^2+^ transient amplitudes and lower SR Ca^2+^ stores that correlated with impaired cardiac function in Sirt1^−/−^ mice; these results were in accordance with that from a previous report.^14^ We suggest the smaller SR Ca^2+^ stores may be attributed to the less Ca^2+^ loading effect from the smaller *I*_Ca,L_ and impaired Ca^2+^ reuptake from the down‐regulated SERCA2a in Sirt1^−/−^ myocytes, leading to smaller Ca^2+^ transient and impaired contractility. Depleted levels of SR Ca^2+^ may also result from reduced function of SERCA2a and increased open probability of Ryanodine receptor 2 (RyR2) to cause more Ca^2+^ leak from the SR.^26^ Calmodulin‐dependent protein kinase II phosphorylates phospholamban at Ser‐10 to decrease the function of SERCA2a.^27^ We speculate that the function of SERCA2a may be further impaired due to phosphorylation of phospholamban by the enhanced levels of CaMKII in Sirt1^−/−^ mice. The Sirt1^−/−^ ventricular myocytes showed increased Ca^2+^ leakage from SR, which would cause diastolic calcium overload. Hyperphosphorylation of RyR2 by CaMKII increases diastolic SR Ca^2+^ leak^28^, ^29^ that may be a reason for the depletion of the SR Ca^2+^ stores and may increase intracellular Ca^2+^ level in Sirt1^−/−^ myocytes. Moreover, the increased reverse‐mode NCX current in Sirt1^−/−^ myocytes may also contribute to the loading of cytosolic [Ca^2+^]. Overloading intracellular Ca^2+^ in cardiomyocytes may trigger ectopic activity, DADs, and, consequently life‐threatening ventricular tachyarrhythmia.^30^ This exacerbates systolic dysfunction and creates a more arrhythmogenic substrate^31^, ^32^ that leads to increased burst firing seen in the ventricles of Sirt1^−/−^ mice. Calmodulin‐dependent protein kinase II were up‐regulated in the Sirt1^−/−^ ventricles. Using KN93, ventricular arrhythmic events were ameliorated. KN93, one of the CaMKII inhibitors, has been shown to have effect against arrhythmias via reduced SR Ca^2+^ leak, Ca^2+^ waves and probabilities of EADs/DADs in various settings of cardiovascular diseases.^33^, ^34^

*I*_Ca,L_ was measured to determine its contribution in the decrease of SR Ca^2+^ content. The current density of *I*_Ca,L_ was lower in Sirt1^−/−^ myocytes compared with that of the control myocytes. This can be attributed to the change in the relative protein levels of L‐type Ca^2+^ channel subunits α1c. The decrease in Ca^2+^ influx via *I*_Ca,L_ implies that the reduced SR Ca^2+^ content levels result from decreased *I*_Ca,L_ in the Sirt1^−/−^ mice. Increased protein levels of NCX may contribute to the increased reverse‐mode NCX current in Sirt1^−/−^ myocytes. While the amount of Ca^2+^ influx increased through reverse‐mode NCX in Sirt1^−/−^ myocytes, which could help to improve the SR Ca^2+^ content, larger Ca^2+^ transient amplitudes, and therefore enhance contractility,^35^ the SR Ca^2+^ loading effect may be compromised resulting in accumulation of cytosolic Ca^2+^ in the face of reduced SERCA2a function, and leaky RYR2.^36^

The amplitudes for *I*_Na, L_ are relatively low but contribute substantially to [Na^+^]*_i_* levels when enhanced due to its slow inactivation characteristics.^37^ *I*_Na,L_ increases in various pathological cardiac conditions, including ischaemia/reperfusion, myocardial infarction and heart failure^38^, ^39^, ^40^ and leads to an overload of intracellular Na^+^ level. Moreover, intracellular Na^+^ homeostasis is tightly connected with Ca^2+^ handling since Na^+^ modulates the operational direction of NCX and increases the diastolic intracellular Ca^2+^ concentration.^41^ Abnormal accumulation of diastolic intracellular Ca^2+^ impairs contractility and is arrhythmogenic.^42^ Increased burst firing in Sirt1^−/−^ mice is abrogated by ranolazine (an *I*_Na,L_ inhibitor). Ranolazine improves the regulation of Ca^2+^ levels and decreases pro‐arrhythmic events by indirectly reducing diastolic Ca^2+^ overload and [Ca^2+^]*_i_* accumulation.^43^, ^44^, ^45^ Increased *I*_Na,L_ results from enhanced activity of CaMKII.^46^, ^47^ Calmodulin‐dependent protein kinase II phosphorylates Na_v_1.5 to regulate its magnitude and other properties, including *I*_Na,L_ inactivation and recovery from inactivation.^46^, ^48^ Enhanced CaMKII negatively affects the regulation of Na^+^ and Ca^2+^ by phosphorylating various target proteins and channels.^26^, ^29^, ^49^, ^50^, ^51^ Furthermore, increased ROS in Sirt1^−/−^ cardiomyocytes increase *I*_Na, L_ and production of ROS that quickly enhances late *I*_Na, L_, thereby stimulating arrhythmogenesis.^52^

Cytosolic and mitochondrial synthesis of ROS increased in Sirt1^−/−^ mice. Sirt1 controls intracellular ROS production by multiple pathways, such as NF‐κB signalling^53^ and affects mitochondrial respiration and, subsequently, ROS production by modulating PGC‐1α activity.^54^ Sirt1 deficiency increases the production of ROS. RyR hyperphosphorylation‐associated Ca^2+^ leak by CaMKII may increase cytosolic levels of Ca^2+^, thereby enabling an overload of mitochondrial Ca^2+^ and facilitating ROS production. Moreover, mitochondria‐derived ROS induces local release of ER Ca^2+^ in cardiomyocytes. ROS‐activated CaMKII enhances *I*_Na,L_ that results in an overload of cellular Na^+^ and enhances Ca^2+^ influx via reversed mode of NCX, thereby enabling arrhythmia.^55^ In this study, we used cardiac‐specific Sirt1 knockout mice to assess whether Sirt1 deficiency alters Ca^2+^ handling in cardiomyocytes. Sirt1^−/−^ mice hearts exhibited increased arrhythmia that was inhibited by KN93 and ranolazine. The Sirt1‐deficient mice showed lower *I*_Ca,L_ and Ca^2^ ^+^ transient together with poor cardiac function. Enhanced *I*_Na,L_ and reversed mode of NCX were observed in Sirt1‐deficient cardiomyocytes with increased production of ROS and CaMKII expression. These findings provide insights into novel mechanisms underlying arrhythmia associated with Sirt1 deficiency.

In conclusion, Sirt1 deficiency in the cardiac tissues resulted in detrimental effects on Ca^2+^ and Na^+^ regulation in mice cardiomyocytes. Dysregulated Ca^2+^ handling and Na^+^ regulation leads to a higher frequency of ventricular arrhythmia and cardiac dysfunction. *I*_Na,L_ was enhanced alongside with increased cytosolic Na^+^ level. ROS production and expression of CaMKII were higher in the Sirt1^−/−^ mice. The CaMKII inhibitor KN93 and ranolazine prevented arrhythmia. These findings suggest that the deficiency of Sirt1 in the cardiomyocytes leads to dysregulation of intracellular Ca^2+^ and Na^+^ that provide proarrhythmic substrates.

## CONFLICT OF INTEREST

The authors declare no conflict of interest.

## AUTHOR CONTRIBUTIONS

H.‐Y. Yang, F.‐Z. Lin and H.‐W. Yang performed experiments; H.‐Y. Yang, F.‐Z. Lin, P.‐L. Yu and S.‐M. Huang analysed data. H.‐Y. Yang, Y.‐C. Chen, C.‐S. Tsai and C.‐Y. Lin interpreted results of experiments; H.‐Y. Yang, F.‐Z. Lin and Y.‐C. Chen prepared figures; H.‐Y. Yang drafted manuscript; H.‐Y. Yang and Y.‐C. Chen edited and revised manuscript; Y.‐C. Chen, C.‐S. Tsai and C.‐Y. Lin conceived and designed research. All authors approved final version of manuscript.

## Data Availability

The data of the present study are available from the corresponding authors following reasonable request.
